# What the eyes say about planning of focused referents during sentence formulation: a cross-linguistic investigation

**DOI:** 10.3389/fpsyg.2014.01124

**Published:** 2014-10-02

**Authors:** Lesya Y. Ganushchak, Agnieszka E. Konopka, Yiya Chen

**Affiliations:** ^1^Leiden University Centre for LinguisticsLeiden, Netherlands; ^2^Education and Child Studies, Faculty of Social and Behavioral Sciences, Leiden UniversityLeiden, Netherlands; ^3^Leiden Institute for Brain and CognitionLeiden, Netherlands; ^4^Max Planck Institute for PsycholinguisticsNijmegen, Netherlands

**Keywords:** focus planning, discourse context, sentence formulation, incrementality, eye-tracking

## Abstract

This study investigated how sentence formulation is influenced by a preceding discourse context. In two eye-tracking experiments, participants described pictures of two-character transitive events in Dutch (Experiment 1) and Chinese (Experiment 2). Focus was manipulated by presenting questions before each picture. In the Neutral condition, participants first heard “*What is happening here?*” In the Object or Subject Focus conditions, the questions asked about the Object or Subject character (*What is the policeman stopping? Who is stopping the truck?*). The target response was the same in all conditions (*The policeman is stopping the truck*). In both experiments, sentence formulation in the Neutral condition showed the expected pattern of speakers fixating the subject character (*policeman*) before the object character (*truck*). In contrast, in the focus conditions speakers rapidly directed their gaze preferentially only to the character they needed to encode to answer the question (the *new*, or *focused*, character). The timing of gaze shifts to the new character varied by language group (Dutch vs. Chinese): shifts to the new character occurred earlier when information in the question can be repeated in the response with the same syntactic structure (in Chinese but not in Dutch). The results show that discourse affects the timecourse of linguistic formulation in simple sentences and that these effects can be modulated by language-specific linguistic structures such as parallels in the syntax of questions and declarative sentences.

## Introduction

To produce a sentence, speakers must prepare a preverbal message and then encode it linguistically. These processes are assumed to proceed incrementally (e.g., Kempen and Hoenkamp, 1987). However, the amount of linguistic information that speakers prepare in advance of speaking can be highly variable (e.g., Konopka, 2012; Konopka and Meyer, 2014). While much work has been done on formulation of individual sentences produced out of context, a largely neglected area of research is how sentences are planned as a function of the discourse context in which they are produced. The aim of the present project is to investigate the timecourse of online sentence formulation within one particular discourse context—i.e., as a function of changes in informational focus.

**Figure 1:**
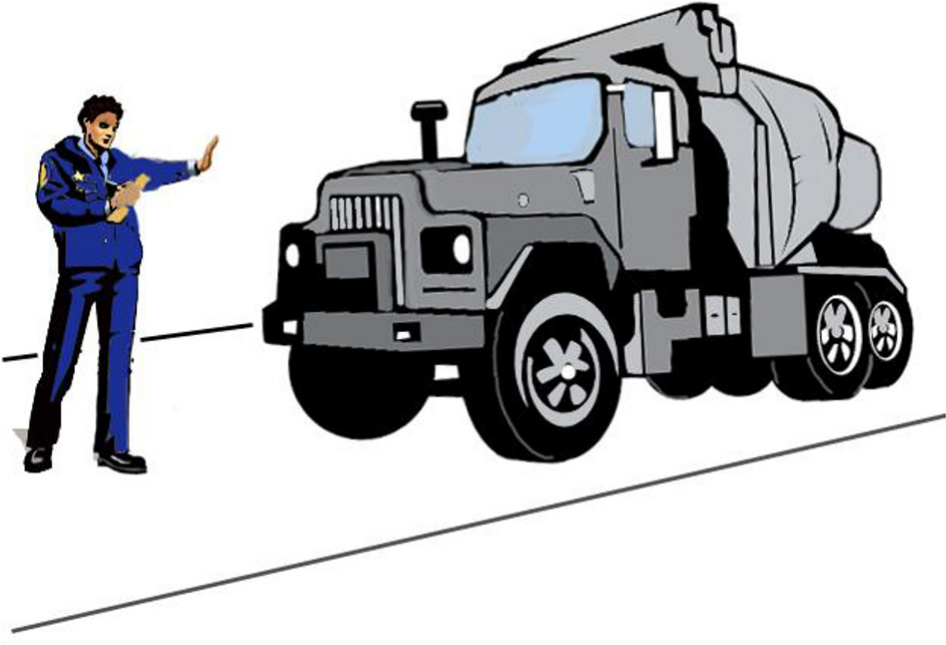
**Example of a target picture event**.

Specifically, we consider formulation of simple event descriptions like *The policeman is stopping the truck* (Figure 1) in response to informational wh-questions. For examples, questions like “*What is the policeman stopping?*” provide a discourse context that establishes one referent in the event as contextually *old* information and the referent that is being asked about as *new*, and therefore *focused*, information (Gussenhoven, 2007). Thus, in answer to this question, the typical answer (*The policeman is stopping the truck*) includes *policemen* as given information and *truck* as new (focused) information. In contrast, if the question is *Who is stopping the truck?*, the typical answer (*The policeman is stopping the truck*) includes *policeman* as the focused referent, indicating that it is the policeman, rather than a person of another profession, who is stopping the *truck*.

The issue we address here is to what extent focus may affect the way utterances are planned online. Sentence formulation is normally investigated by asking speakers to describe pictures of events (Figure 1) while their gaze and speech are recorded (Griffin and Bock, 2000; Bock et al., 2004; Griffin, 2004; Meyer and Lethaus, 2004; Gleitman et al., 2007; Kuchinsky and Bock, 2010; Konopka, 2013, 2014; Ganushchak et al., 2014; Konopka and Meyer, 2014; Van de Velde et al., 2014). On Griffin and Bock's (2000) account, formulation begins with an apprehension phase (0–400 ms after picture onset) during which speakers encode the “gist” of the event. During this phase, fixations to the subject and object characters in the event do not differ from each other reliably. Event apprehension is then followed by a longer phase of linguistic encoding that lasts until the end of articulation. In this time window (400 ms until the end of speech), participants normally look at characters in the display in the order of mention. Viewing times on a character and gaze shifts from one character to another after 400 ms are thus expected to vary with the ease of encoding each character (e.g., easy-to-name characters are fixated for less time than harder-to-name characters; see Konopka and Meyer, 2014; Van de Velde et al., 2014).

To compare formulation of sentences with and without focus, eye-tracked participants were asked to describe pictures shown on a computer screen in their native language: Dutch (Experiment 1) or Chinese (Experiment 2). Focus was manipulated by means of questions that preceded each picture. In the Neutral condition, participants were asked a question that was neutral with respect to discourse focus: “*What is happening here*?” In the remaining two conditions, the questions changed the discourse focus of the expected target event description. In the Subject Focus condition, participants were asked about the subject character (*Who is stopping the truck?*). In the Object Focus condition, participants were asked about the object character (*What is the policeman stopping*?). The expected target response had the same structure and content in all conditions (*The policeman is stopping the truck*).

How might discourse focus influence formulation? Differences in planning of the target responses were evaluated by comparing speakers' eye movements to the two event characters prior to speech onset. On the one hand, it is possible that discourse focus does not immediately influence the timecourse of formulation. If so, viewing times for the subject and object characters should not differ across conditions: speakers should consistently fixate the subject character first and then direct their attention and gaze to the object character, reflecting order of mention. This outcome would be expected on the basis of research showing very tight gaze-speech coordination during formulation (e.g., Griffin and Bock, 2000), even when speakers talk about “old” or previously inspected referents (e.g., Meyer et al., 2004). On the other hand, if sentence formulation is sensitive to changes in information structure at the discourse level, then changes in the *old/new* (or *focused/unfocused*) status of event characters should influence the relative allocation of attention to these characters. In this case, viewing patterns in the Subject and Object focus conditions should differ from the Neutral Focus condition: speakers should direct fewer fixations to the character that was mentioned in the question (the *old* character) but should preferentially fixate the character needed to answer the question (the *new*, or *focused*, character). Thus, in the Object Focus condition, speakers should rapidly direct their gaze to the object character, and in the Subject Focus condition, they should direct their gaze to the subject character.

We also test whether changes in gaze patterns are modulated exclusively by discourse context or if they also depend on the ease of encoding the target sentences linguistically. The questions in the Object and Subject Focus conditions mention one of the event characters, which establishes this character as *old* information in the discourse and provides speakers with a referential term they can use in their responses. Thus, by definition, the questions in the Focus conditions facilitate conceptual and linguistic planning of the *old* character. However, in addition to recognizing the *old* character in the event, speakers must also generate a suitable sentence structure to produce a full response to the preceding question. To test whether formulation additionally depends on the ease of linguistic encoding in the Focus conditions, Experiments 1 and 2 compare sentence formulation in the same task with speakers of two languages that differ in the word order of wh-questions: Dutch and Chinese. Dutch requires *wh*-fronting (*Who is stopping the truck? What is the policeman stopping?*), while Chinese is known for *in-situ wh*-questions (i.e., *wh*-words do not undergo movement but remain in the same surface syntactic position as the constituent being question; Cheng, 2009). This is illustrated in the following examples:

Subject focus: 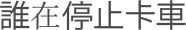 (*Who is stopping the truck?*)
Object focus: 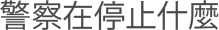 (The policeman is stopping what?)

So, the two languages have the same surface word order when the focus of the *wh*-question is on the subject character but very different orders when the focus of the *wh*-question is on the object character. Consequently, when prompted by an object-specific *wh*-question (i.e., Object Focus question), Chinese speakers are provided with linguistic material that they can repeat verbatim in their response without having to change the syntactic constituent order provided in the *wh*-question, while Dutch speakers need to generate a response with a word order different from that of the preceding question. If sentence formulation is sensitive to the amount of information provided in the preceding discourse context even at the syntactic structural level, we should observe a cross-linguistic difference in sentence formulation after Object Focus questions in Experiment 1 (Dutch) and Experiment 2 (Chinese): since Chinese speakers can “reuse” linguistic material from the question without syntactic restructuring when preparing their response, they may begin shifting their gaze to the *new* object character earlier than speakers of Dutch (who, besides encoding the object character, must also generate a suitable sentence structure).

Importantly, we test how early differences in fixation patterns to the subject and object characters emerge in the Object and Subject focus conditions compared to the Neutral condition. Overall, differences occurring immediately after picture onset (0–400 ms, i.e., a window arguably corresponding to event apprehension) would indicate that focus information has an early effect on formulation of the target utterance—beginning during the encoding of the preverbal message. In contrast, differences across conditions emerging after 400 ms would indicate that focus information influences primarily the timing of linguistic encoding, after speakers have encoded the gist of the event they are about to describe.

## Experiment 1. focus planning: Dutch

### Methods

#### Participants

Thirty native speakers of Dutch, all students at Leiden University, participated in the experiment (24 women; age range 17–23 years). All participants were students at Leiden University. The study was conducted in accord with APA standards for ethical treatment of participants and was approved by the ethical committee board of Leiden University. Participants gave written informed consent prior to participating and received a small financial reward.

#### Materials

The stimulus lists consisted of 178 colored pictures displaying simple events (Figure 1). There were 58 target pictures of transitive events, 116 fillers, and 4 practice pictures. In the target pictures, the subject character was on the left in 77% of the cases^1^. Discourse focus was manipulated by means of questions presented before each picture.

- Neutral question:
  *Wat gebeurt hier?* (What is happening here?)
- Object Focus question:
  *Wat stopt de politieman?* (What is the policeman stopping?)
- Subject Focus question:
  *Wie stopt de vrachtauto?* (Who is stopping the truck?)

Modal target sentence: *De politieman laat een vrachtauto stoppen* (The policeman is stopping the truck).

All questions were recorded by a native Dutch male speaker and were presented auditorily prior to picture onset.

#### Design and procedure

Lists of stimuli were created to counterbalance question type across target pictures. Each target picture occurred in Focus condition on different lists, so each participant saw each picture only once.

Target pictures were interspersed among filler pictures, with at least two filler pictures separating any two target trials in each list. The fillers showed similar one-character and two-character events. However, the questions preceding filler pictures varied: e.g., the questions asked participants to name the color of an object, or to count how many of a given item appeared in the picture.

Participants were seated in a sound-proof room. Eye movements were recorded with an Eyelink 1000 eye-tracker (SR Research Ltd.; 500 Hz sampling rate). Eye calibration was done at the beginning of the experiment, using a 9-point calibration procedure. Participants first heard a question (presented through headphones). Experimenter then clicked with the mouse after completion of the question to proceed to the picture trials. Picture trials began with a fixation point presented at the top of the screen (drift correction): participants had to fixate the fixation point and press the space bar to display the picture. They were instructed to describe each picture with one sentence and were not under time pressure to produce the response. The experimenter clicked with the mouse when the participant finished speaking. On average, the pictures were displayed on the screen for 4191 ms (*SD* = 850 ms). The task started with four practice trials.

#### Scoring and data analysis

Target sentences were scored as correct if participants used an active SVO structure. Trials where participants used a different structure (e.g., passive sentences) or made corrections during the description were excluded from analysis (7% of the data; Subject Focus: 1.1%; Object Focus: 1.4%; Neutral: 4.6%; error rates were lower than in other reported studies, largely because the experimental manipulations successfully constrained structure choice on target trials to SVO sentences).

Interest areas were drawn around each character in the target pictures (allowing a 2–3 cm margin around each character). Trials in which the first fixation was within the subject or object character interest area instead of the fixation point were also removed from the analyses (1% of the data). This left 883 trials for analysis.

Analyses were carried out a) on speech onsets to assess differences across conditions with respect to encoding difficulty in sentences with *new* and *old* subject and object characters, and b) on subject-directed fixations to assess differences in the timecourse of formulation across conditions.

Speech onsets were first log-transformed to remove the intrinsic positive skew and non-normality of the distribution, and then submitted to mixed-effects model analyses with participants and items as random effects (Baayen et al., 2008). Focus Location (Neutral, Object Focus, and Subject Focus) was entered as a fixed effect. By-subject and by-item random slopes for Focus Location and random intercepts were also included. Onsets in the three Focus Location conditions were compared with two contrasts using treatment coding. The first contrast compared the Neutral condition against the Object Focus condition; the second contrast compared the Neutral condition against the Subject Focus condition. Both contrasts thus assess how planning a sentence in response to a question that mentions one of the event characters changes response latencies relative to the neutral condition. Next, a separate analysis was run with new contrasts to compare response latencies in the Subject and Object Focus conditions against one another.

For the timecourse analyses, the distribution of subject-directed fixations in sentences produced in the three conditions was compared with by-participant (β_1_) and by-item (β_2_) quasi-logistic regressions (Barr, 2008). Consistent with earlier work and based on visual inspection of the distributions, we selected three time windows (0–400, 400–800, and 800–1600 ms) for analysis. The first time window arguably corresponds to a period of event apprehension (Griffin and Bock, 2000; Konopka and Meyer, 2014), while the second and third time windows include the rise and fall of fixations to the subject character before speech onset in the Neutral condition (within each of these windows, changes of fixation proportions show a relatively linear pattern as a function of time). Fixations were aggregated into a series of 200 ms time bins for each participant in the by-participant analysis and each item in the by-item analysis in each condition. The dependent variable in each time bin was an empirical logit indexing the likelihood of speakers fixating the subject characters out of the total number of fixations observed in that time bin.

The models included Time Bin and Focus Location (Neutral, Subject Focus, and Object Focus) as fixed effects, and tested for interactions between these variables. All models included random by-participant and by-item random intercepts and slopes for the Time and Focus Location variables. For interactive models, the random effects structure included the interaction between Time and Focus Location; in additive models, the models included additive random slopes for Time and Focus Location. Main effects in these analyses indicate differences across conditions in the first bin of each window, while interactions with Time show how fixation patterns changed over the remaining bins in that time window. Thus, when we refer to an effect (a main effect) present at 0–200, 400–600, or at 800–1000 ms, we are describing a difference between conditions present at the first 200 ms of a time window. Interactions between the Focus Location factor and the Time factor then show how the pattern of fixations changed in the remaining time window (200–400, 600–800, and 1000–1600 ms, respectively). The log-likelihood ratio test (χ^2^) was used to compare model fit in interactive and additive models, and thus test whether interactions with the Time variable significantly improved model fit (a reliable difference in this comparison indicates a better fit for the interactive model than the additive model). All interactions reported below were reliable by this criterion at *p* < 0.01.

As in the analyses of speech onsets, fixations in the three Focus Location conditions were compared with two contrasts, and the Object and Subject Focus conditions were compared against each other in a separate analysis.

### Results

#### Speech onsets

Participants started speaking significantly later in the Neutral condition than in the Object and Subject focus conditions (β = −0.24, *SE* = 0.04; *t* < −6; β = −0.17, *SE* = 0.04; *t* < −4), for the two contrasts respectively; see Table 1 for means). The difference in speech onset latencies between the Object Focus and Subject Focus conditions was not significant (*t* < 1).

**Table 1:** **Mean response latencies in ms (and standard errors) per condition in Experiment 1 (Dutch) and in Experiment 2 (Chinese)**.

	**Object focus**	**Subject focus**	**Neutral**
Experiment 1	1550 (412)	1555 (265)	2104 (623)
Experiment 2	1139 (452)	1610 (735)	1822 (588)

#### Timecourse of sentence formulation

**Figure 2:**
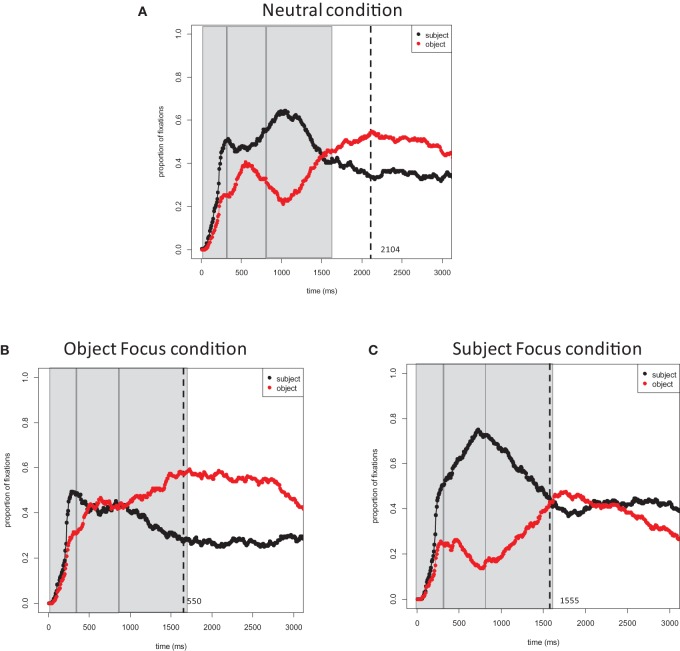
**Experiment 1 (Dutch)**. Proportions of fixations to the subject and object characters in target event pictures: **(A)** Neutral Focus condition (*Wat gebeurt hier?*; *What is happening?*); **(B)** Object Focus condition (*Wat stopt de politieman?; What is the policeman stopping?*); **(C)** Subject Focus condition (*Wie stopt de vrachtauto?*; *Who is stopping the truck?*). Time 0 corresponds to picture onset. Dashed lines represent speech onsets. Areas selected by rectangles depict the three time window (0–400, 400–800, and 800–1600 ms) used in the analyses.

Figure 2 plots the proportions of fixations to the subject and object characters in target pictures across conditions. **Figure 4A** then plots the proportions of fixations to the subject character in the target pictures across all three conditions. Results of all timecourse analyses are listed in Table 2 (the by-participants and by-items analyses provided largely converging results and are thus not discussed separately).

**Table 2:** **Results of by-participant (β_1_) and by-item (β_2_) quasi-logistic regressions carried out over three time windows in Experiment 1 (Dutch) and Experiment 2 (Chinese)**.

	**Experiment 1**		**Experiment 2**	
	**Main effect of Focus location**	**Interaction of Focus Location with Time**	**Main effect of Focus location**	**Interaction of Focus Location with Time**
**(A) 0–400 ms**				
1. Neutral vs. Object Focus	β_1_ = −0.12, *SE* = 0.24, *t* = −0.51	β_1_ = −0.32, *SE* = 0.91, *t* = −0.35	β_1_ = −0.13, *SE* = 0.21, *t* = −0.61	β_1_ = 0.07, *SE* = 0.74, *t* = 0.09
	β_2_ = −0.15, *SE* = 0.16, *t* = −0.92	β_2_ = −0.01, *SE* = 0.62, *t* = −0.01	β_2_ = −0.07, *SE* = 0.17, *t* = −0.42	β_2_ = −0.10, *SE* = 0.59, *t* = −0.17
2. Neutral vs. Subject Focus	β_1_ = −0.16, *SE* = 0.24, *t* = −0.69	β_1_ = 1.26, *SE* = 0.88, *t* = 1.43	β_1_ = −0.37, *SE* = 0.21, *t* = −1.79^†^	β_1_ = 0.85, *SE* = 0.77, *t* = 1.11
	β_2_ = −0.12, *SE* = 0.16, *t* = −0.72	β_2_ = 1.17, *SE* = 0.61, *t* = 1.91^†^	β_2_ = −0.27, *SE* = 0.18, *t* = −1.63	β_2_ = 0.86, *SE* = 0.64, *t* = 1.33
3. Object vs. Subject Focus	β_1_ = −0.04, *SE* = 0.21, *t* = −0.17	β_1_ = 0.86, *SE* = 0.70, *t* = 1.22	β_1_ = −0.12, *SE* = 0.17, *t* = −0.71	β_1_ = 0.38, *SE* = 0.61, *t* = 0.62
	β_2_ = 0.01, *SE* = 0.14, *t* = 0.09	β_2_ = 0.62, *SE* = 0.55, *t* = 1.13	β_2_ = −0.10, *SE* = 0.13, *t* = −0.76	β_2_ = 0.47, *SE* = 0.49, *t* = 0.95
**(B) 400–800 ms**				
1. Neutral vs. Object Focus	β_1_ = 0.57, *SE* = 0.30; *t* = 1.91^†^	β_1_ = −1.91, *SE* = 0.47; *t* = −4.07^***^	β_1_ = 1.16, *SE* = 0.42, *t* = 2.74^**^	β_1_ = −3.23, *SE* = 0.70, *t* = −4.59^***^
	β_2_ = 0.64, *SE* = 0.29; *t* = 2.18^*^	β_2_ = −2.01, *SE* = 0.48; *t* = −4.17^***^	β_2_ = 1.14, *SE* = 0.35, *t* = 3.27^***^	β_2_ = −3.21, *SE* = 0.58, *t* = −5.50^***^
2. Neutral vs. Subject Focus	β_1_ = −0.03, *SE* = 0.33; *t* = −0.09	β_1_ = 1.53, *SE* = 0.48; *t* = 3.21^***^	β_1_ = −1.12, *SE* = 0.39, *t* = −2.90^**^	β_1_ = 3.34, *SE* = 0.63, *t* = 5.30^***^
	β_2_ = −0.12, *SE* = 0.29; *t* = −0.41	β_2_ = 1.73, *SE* = 0.47; *t* = 3.68^***^	β_2_ = −1.14, *SE* = 0.43, *t* = −2.64^**^	β_2_ = 3.44, *SE* = 0.68, *t* = 5.05^***^
3. Object vs. Subject Focus	β_1_ = −0.31, *SE* = 0.26, *t* = −1.19	β_1_ = 1.74, *SE* = 0.38, *t* = 4.53^***^	β_1_ = −1.15, *SE* = 0.34, *t* = −3.38^***^	β_1_ = 3.29, *SE* = 0.57, *t* = 5.80^***^
	β_2_ = −0.36, *SE* = 0.23, *t* = −1.59	β_2_ = 1.84, *SE* = 0.38, *t* = 4.84^***^	β_2_ = −1.14, *SE* = 0.31, *t* = −3.66^***^	β_2_ = 3.31, *SE* = 0.50, *t* = 6.60^**^
**(C) 800–1600 ms**				
1. Neutral vs. Object Focus	β_1_ = −1.60, *SE* = 0.35, *t* = −4.57^***^	β_1_ = 0.63, *SE* = 0.29; *t* = 2.18^*^	β_1_ = −1.93, *SE* = 0.46, *t* = −4.14^***^	β_1_ = 0.54, *SE* = 0.39, *t* = 1.40
	β_2_ = −1.63, *SE* = 0.32, *t* = −5.13^***^	β_2_ = 0.70, *SE* = 0.22; *t* = 2.76^**^	β_2_ = −1.93, *SE* = 0.47, *t* = −4.14^***^	β_2_ = 0.54, *SE* = 0.37, *t* = 1.40
2. Neutral vs. Subject Focus	β_1_ = 1.60, *SE* = 0.32; *t* = 5.08^***^	β_1_ = −0.84, *SE* = 0.27; *t* = −3.14^**^	β_1_ = 2.19, *SE* = 0.39, *t* = 5.54^***^	β_1_ = −1.2, *SE* = 0.29, *t* = −3.76^***^
	β_2_ = 1.67, *SE* = 0.25; *t* = 5.91^***^	β_2_ = −0.92, *SE* = 0.22; *t* = −4.17^***^	β_2_ = 2.19, *SE* = 0.40, *t* = 5.54^***^	β_2_ = −1.12, *SE* = 0.29, *t* = −3.76^***^
3. Object vs. Subject Focus	β_1_ = 1.60, *SE* = 0.27; *t* = 5.91^***^	β_1_ = −0.74, *SE* = 0.23; *t* = −3.22^***^	β_1_ = 2.04, *SE* = 0.34, *t* = 5.92^***^	β_1_ = −0.81, *SE* = 0.27, *t* = −2.99^**^
	β_2_ = 1.66, *SE* = 0.24; *t* = 6.92^***^	β_2_ = −0.81, *SE* = 0.19; *t* = −4.23^***^	β_2_ = 2.03, *SE* = 0.34, *t* = 5.92^***^	β_2_ = −0.81, *SE* = 0.27, *t* = −2.99^**^

*The table lists statistics for the main effects of the individual Focus comparisons and for interactions of the Focus condition with Time (all main effects for the Time variable were reliable, and are thus not listed). Two analyses were carried out on subject-directed fixations within each time window: the first two contrasts (1, 2) show results from the analysis performed with Focus Location as a three-factor variable (Neutral, Object, Subject), while the third contrast (3) shows results from the analysis performed with Focus Location as a two-factor variable (Object, Subject). Significance for individual effects was determined by treating t-values as if they were drawn of the normal distribution (see Barr, 2008)*.

† *p < 0.10*.

* *p < 0.05*.

** ^**^p < 0.01.

*** *p < 0.001*.

***0–400 ms***. In all conditions, speakers rapidly directed their gaze to the subject character in the event within 400 ms of picture onset. All main effects and interactions in this time window did not reach significance (Table 2A).

***400–800 ms***. After 400 ms, speakers largely directed their gaze to the subject character in the Neutral condition. The first contrast in this analysis showed a weak difference in fixations to subject characters at the first time bin (i.e., 400–600 ms) in the Neutral condition and Object Focus condition (the effect was reliable in the by-item analysis). The interaction between Focus Location and Time was reliable: in the Neutral condition, speakers quickly directed their gaze to the subject character while in the Object focus condition, fixations to the subject character remained stable. The second contrast in the analysis showed that fixations to the subject character did not differ in the Neutral condition and Subject Focus condition at 400–600 ms. The interaction with Time for this contrast was again significant: speakers directed their gaze preferentially to subject characters in the Subject Focus condition while fixations to subject characters remained stable in the Neutral condition (Table 2B).

Comparing the Subject Focus and Object Focus conditions against one another in a separate analysis showed a significant interaction of Focus Location with Time. Thus, as time progressed, fixations to the subject character within this window increased in the Subject Focus condition but not in the Object Focus condition.

***800–1600 ms***. Speakers began shifting their gaze away from the subject character between 800 ms and speech onset. Carrying over from earlier windows, speakers were more likely to fixate subject characters in the Neutral condition than in the Object Focus condition during the first 200 ms of the time window (i.e., 800–1000 ms), but were more likely to fixate subject characters in the Subject Focus condition than in the Neutral condition. The first contrast in the interaction between Time and Focus Location was significant, showing that fixations to the subject character decreased at a steeper rate in the Object Focus condition than in the Neutral condition. The second contrast in this interaction was also significant: fixations to subject characters decreased at a steeper rate in the Neutral condition than in the Subject Focus condition (Table 2C).

Finally, the comparison between Subject Focus and Object Focus conditions showed that there were more fixations to subject characters in the Subject Focus condition than in the Object Focus condition at the first 200 ms of the time window (i.e., 800–1000 ms). The interaction with Time was also significant: fixations to subject characters decreased at a steeper rate in the Subject Focus condition than in the Object Focus condition.

### Discussion

Speakers' gaze patterns showed large differences in attention allocation to subject and object characters in target events across conditions. The pattern obtained in the Neutral condition replicated earlier findings, showing that participants largely fixate characters in the order of mention: first the subject character (*policeman*) and then the object character (*truck*; Griffin and Bock, 2000). Gaze shifts to the object character occurred well before speech onset.

In contrast, sentence formulation in the Subject Focus and Object Focus conditions was strongly influenced by the preceding discourse context. First, speech onsets were reliably shorter in these conditions than in the Neutral condition, suggesting that partial knowledge of the characters and of the relationship between characters in the upcoming event facilitated planning. Second and more importantly, the distribution of fixations to the two characters across conditions was strongly influenced by the preceding discourse questions. Speakers had a strong preference for fixating the contextually *new* character with priority, both when this character was the sentence subject and when it was the sentence object. In the Object Focus condition, participants looked briefly at the subject character and shifted their gaze to the object character shortly after 400 ms of the picture onset, while in the Subject Focus condition, participants looked longer at the subject character and shifted their gaze to the object character only about 1600 ms after picture onset. Thus, even though the propositional content and the surface form of the target sentence were held constant across conditions, gaze-speech coordination during sentence formulation changed with discourse context.

## Experiment 2. focus planning: Chinese

### Methods

#### Participants

Thirty native speakers of Chinese (Northern regions) participated in the experiment (16 women; age range 23–29 years). All participants were students at Leiden University. Research reported in the current manuscript was conducted in accord with APA standards for ethical treatment of participants and was approved by the ethical committee board of Leiden University. Participants gave written informed consent prior to participating in the study and received a small financial reward after the experiment.

#### Materials

The pictures used in this experiment were a subset of the pictures described in Experiment 1. Fifteen target pictures were excluded as they were unlikely to elicit SVO descriptions in Chinese. Thus, in total, there were 129 colored pictures in Experiment 2 (43 target pictures, 82 fillers, and 4 practice pictures). In the target pictures, the subject character was on the left in 74% of the cases. As in Experiment 1, focus was manipulated by means of questions that preceded each picture. All questions were recorded by a native Chinese female speaker.

#### Design, procedure, and data analysis

The design, procedure and analyses were identical to Experiment 1. The target pictures remained on the screen for about 4541 ms (*SD* = 856 ms). In total, 11% (Subject Focus: 2.6%; Object Focus: 3.3%; Neutral: 4.8%) of all target trials were removed due to erroneous responses and 1% of trials removed because the first fixation was within the subject or object character interest area instead of the fixation point. This left 527 trials for analysis.

### Results

#### Speech onsets

Participants started speaking significantly later in the Neutral condition than in the Object Focus conditions (β = −0.56, *SE* = 0.07; *t* < −8; see Table 1 for means). The difference in speech onset latencies between the Neutral and Subject Focus conditions was not significant (*t* < 1.5). Participants also started speaking later in the Subject Focus conditions than in the Object Focus conditions (β = 0.34, *SE* = 0.05; *t* > 6).

#### Timecourse of formulation

**Figure 3:**
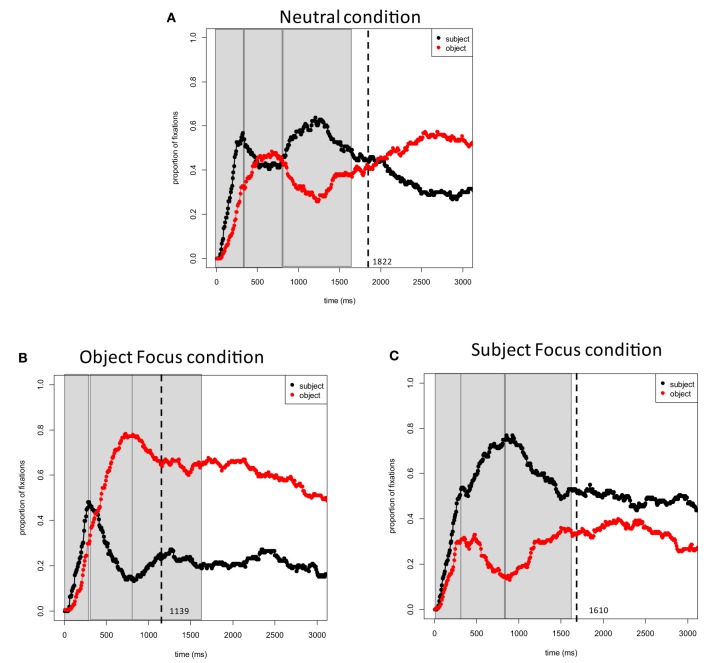
**Experiment 2 (Chinese)**. Proportions of fixations to the subject and object characters in target event pictures: **(A)** Neutral Focus condition (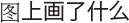; *What is happening?*); **(B)** Object Focus condition (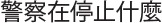; *The policeman is stopping what*?); **(C)** Subject Focus condition (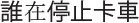; *Who is stopping the truck?*). Time 0 corresponds to picture onset. Dashed lines represent speech onset. Areas selected by rectangles depict the three time windows (0–400, 400–800, and 800–1600 ms) used in the analyses.

**Figure 4:**
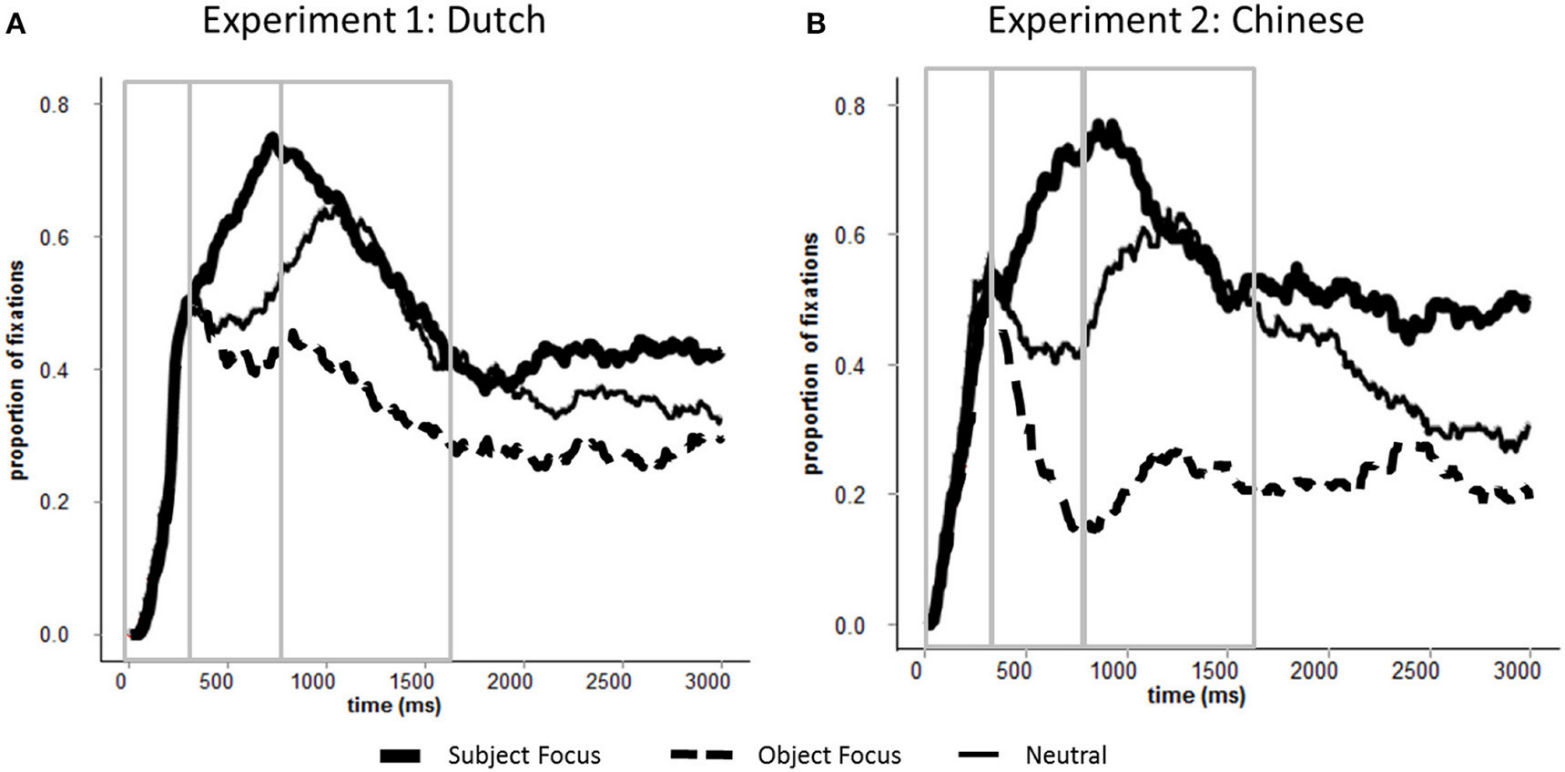
**Proportions of fixations to the subject characters in target event pictures across all conditions (A) Experiment 1 (Dutch); (B) Experiment 2 (Chinese)**. Time 0 corresponds to picture onset. Areas selected by rectangles depict the three time windows (0–400, 400–800, and 800–1600 ms) used in the analyses.

Figure 3 plots the proportions of fixations to the subject and object characters in target pictures across conditions. Figure 4B again plots the proportions of fixations to the subject character in the target pictures across all three conditions. The overall distribution of fixations to the two characters was similar to Experiment 1, with the exception of the Object focus condition. Results of statistical tests are provided in Table 2.

***0–400 ms***. In all conditions, speakers rapidly directed their gaze to the subject character in the picture within 400 ms of picture onset. All main effects and interactions in this time window were not significant (Table 2A).

***400–800 ms***. Speakers were already more likely to fixate subject characters in the Object Focus condition than in the Neutral condition at the first 200 ms of the time window (i.e., 400–600 ms), which, in turn, had more fixation than in the Subject Focus condition. All interactions with Time were largely consistent with Experiment 1. The first contrast in the interaction between Focus Condition and Time was significant: fixations to subject characters decreased at a steeper rate in the Object Focus condition than in the Neutral condition. The second contrast in the interaction between Focus Location and Time was also significant: fixations to subject characters decreased in the Neutral condition but increased in the Subject Focus condition (Table 2B).

Comparing the Subject Focus and Object Focus conditions against one another in a separate analysis showed that initially (400–600 ms), speakers fixated subject characters more often in the Subject Focus condition than in the Object Focus condition. As time progressed, speakers also directed their gaze to subject characters in the Subject Focus condition and away from the subject characters in the Object Focus condition (resulting in an interaction of Focus Location with Time).

***800–1600 ms***. In the Neutral condition, speakers briefly directed their gaze to the subject character and then shifted their gaze away from this character between 800 and 1600 ms. In contrast, fixations in the Object and Subject Focus conditions were largely consistent with Experiment 1. Specifically, at the first 200 ms of the time window (i.e., 800–1000 ms), speakers were more likely to fixate subject characters in the Neutral condition than in the Object Focus condition, but were more likely to fixate subject characters in the Subject Focus condition than Neutral condition. The first contrast in the interaction between Focus Location and Time was not significant; the second contrast in this interaction was significant (Table 2C). Interactions with the Time variable are difficult to interpret because of non-linearities in the distribution of fixations in the Neutral condition. Thus for a rough comparison of fixations in this time window across conditions, a complementary analysis was carried out using average empirical logits calculated across the entire time window (i.e., the overall likelihood of speakers fixating the subject character) as the dependent variable. This comparison showed the expected pattern: speakers were more likely to fixate subject characters in the Neutral condition than in the Object Focus condition (β_1_ = −1.28, *SE* = 0.15, *t* = −8.46; β_2_ = −1.31, *SE* = 0.12, *t* = −11.08) and were more likely to fixate subject characters in the Subject Focus condition than in the Neutral condition (β_1_ = 0.75, *SE* = 0.13, *t* = 5.56; β_2_ = 0.75, *SE* = 0.12, *t* = 6.33).

Finally, the Subject Focus and Object Focus conditions were compared against one another. As expected, the analysis showed that speakers were more likely to fixate the subject character in the Subject Focus condition than in the Object Focus condition at the first 200 ms of the time window (i.e., 800–1000 ms). The interaction with Time was also significant: fixations to subject characters decreased steeply in the Subject Focus condition but remained relatively stable in the Object Focus condition.

### Discussion

Experiment 2 replicates the main findings of Experiment 1. First, speech onsets were longer in the Neutral condition than in the Object and Subject Focus conditions. The reduction in speech onset times was largest in the Object Focus condition^2^. Second, and more importantly, Experiment 2 (Chinese) showed strong effects of the preceding discourse context on formulation. The pattern obtained in the Neutral condition again showed that participants looked at event characters in the order of mention, but in the Subject and Object Focus conditions, fixations to the two characters were strongly influenced by the preceding questions: after 400 ms, speakers preferentially and rapidly fixated the contextually *new* character.

Experiment 2 also shows the predicted cross-linguistic difference between Dutch and Chinese. Namely, shifts of gaze to the object character in the Object Focus condition began earlier than in Experiment 1: fixations to the object character increased immediately after 400 ms in Experiment 2 but only after 800 ms in Experiment 1 (see Table 2B for a comparison between experiments). To verify this finding, we ran additional analyses combining data from both experiments. The models included Time Bin, Focus Location (Neutral, Subject Focus, and Object Focus) and Language (Chinese and Dutch) as fixed effects. The analyses showed significant three-way interactions between these factors in the 400–800 ms time window (Neutral vs. Object Focus: β_1_ = 3.08, *SE* = 1.20, *t* = 2.56; β_2_ = 2.15, *SE* = 0.99, *t* = 2.18; Neutral vs. Subject Focus: β_1_ = −2.39, *SE* = 1.11, *t* = −2.14; β_2_ = −1.78, *SE* = 0.95, *t* = −1.87). As outlined earlier, this difference may be due to the fact that the surface word order in the Object Focus questions in Chinese provides speakers with a sentence preamble that they can repeat verbatim in their response: availability of this material may have allowed Chinese speakers to direct their attention to the contextually new character earlier than Dutch speakers were able to do^3^. Consistent with this interpretation is also the large difference in speech onsets between the Object Focus and Subject Focus conditions in Experiment 2 (approximately 470 ms; this difference was only 5 ms in Experiment 1): Object Focus responses to questions in Chinese may have been easiest to prepare because speakers could repeat linguistic material from the question.

## General discussion

Two experiments compared the timecourse of formulation for sentences produced in response to three types of questions in Dutch and Chinese. The questions either provided no discourse context for the target event (Neutral condition) or specifically asked about one of the event characters (Object and Subject Focus conditions). The results showed that questions did not influence the distribution of attention to the two event characters immediately after picture onset (0–400 ms), i.e., during a period of message-level encoding. However, the highly linear pattern of formulation observed in the Neutral condition after 400 ms (e.g., Griffin and Bock, 2000; Konopka and Meyer, 2014) was different after Object Focus and Subject Focus questions: instead of fixating characters in the order of mention, speakers fixated primarily the *new* character, regardless of its position in the sentence.

Differences in the likelihood of speakers fixating the subject and object characters in the Neutral condition and the two Focus conditions can be attributed to at least two factors. First, questions provided a discourse context that either did not draw attention to the subject and object characters (Neutral condition) or that did explicitly require preferential encoding of the contextually *new* character (Focus conditions). Second, explicit mention of one character in the question reduced the costs of retrieving its name when describing the target event and thus reduced the likelihood of speakers fixating this character (also see Konopka, 2014). Experiment 2 showed that reducing the costs of generating the target sentence itself in Chinese further reduced the likelihood of speakers fixating the old character.

The observed difference between Dutch and Chinese across experiments lends convincing evidence that sentence planning can be influenced by the linguistic context in which a target utterance is prepared and produced. Differences in the grammaticalized word orders in Chinese and Dutch facilitated production in Chinese as Chinese speakers could start by repeating verbatim the subject and verb of the preceding question without any further re-ordering of the syntactic constituents as is necessary for Dutch. The cross-linguistic difference therefore may be partly due to repetition priming and syntactic priming (e.g., Pickering and Branigan, 1998, 1999): given the compatible word order in the Object Focus question and the response in Chinese, priming is possible for Chinese speakers but not for Dutch speakers. To the extent that eye movements provide insight into the allocation of attention and resources to different encoding processes, large changes in the temporal coupling of gaze and speech suggest that context can strongly influence the incremental formulation of simple utterances. Specifically, the results of both experiments show strong effects of top–down guidance from the message level and contextual facilitation of linguistic encoding: on the basis of their encoding of event gist immediately after picture onset (0–400 ms) and their exposure to linguistic material in the question, speakers deployed their gaze only to the character they needed to encode to answer the question. Thus, eye movements in the Object and Subject Focus conditions show that shifts of gaze need not closely reflect the order of linguistic encoding operations. Rather, they are better indicators of *higher-level* communicative goals and recent linguistic experience: speakers direct their attention to whatever part of the display they need to process with priority to produce a contextually fitting response. Tight coordination of gaze and speech (e.g., Griffin and Bock, 2000) may therefore be more representative of formulation of sentences out of context, where all information in a to-be-described event is new and unfocused.

More generally, the results are compatible with theories of incrementality in sentence formulation that propose top–down guidance during the formulation process (Bock et al., 2004; Konopka and Meyer, 2014; see Gleitman et al., 2007, for an alternative, bottom-up account of sentence formulation). The key assumption of these theories is that sentence formulation begins with the formulation of a message-level representation that guides all subsequent encoding operations, as reflected in the ensuing pattern of eye movements to different parts of a to-be-described event. The results of the current experiments show that, when message-level representations include information about discourse focus, the timecourse of sentence formulation changes immediately to reflect changes in speakers' communicative goals. The high degree of similarity in the timecourse of formulation across languages shows language-general adaptations in the incremental preparation of simple sentences.

### Conflict of interest statement

The authors declare that the research was conducted in the absence of any commercial or financial relationships that could be construed as a potential conflict of interest.

## Footnotes

^1^We cannot say for sure whether the effects in the Neutral condition are due to “order of mention” or to a general left-to-right scanning preference. In the current study, we saw a stronger tendency for speakers to fixate the two characters in the order of mention when the agent appeared on the left hand-side of the screen. However, by comparison, we see very strong effects of the question manipulation on formulation. It is also important to note that all pictures appeared in all of the conditions, so the differences we see between conditions cannot be attributed to the agent placement.

^2^Note that speech onset latencies were somewhat different for Chinese and Dutch speakers. Specifically, Chinese speakers were overall faster than the Dutch participants. Chinese speakers were also faster in initiating speech in the Object Focus condition than the Subject Focus condition, while for Dutch speakers there was no reliable difference in speech onsets in these conditions. We compared speech onsets across the two groups in a complementary analysis with Focus Location (Neutral, Object Focus, Subject Focus) and Language (Chinese vs. Dutch) as fixed effects. The analysis showed a significant interaction between Focus Location and Language (Neutral vs. Object Focus: β = 0.31, *SE* = 0.07, *t* = 4.48; Neutral vs. Subject Focus: β = −0.25, *SE* = 0.06, *t* = −3.58; Object Focus vs. Subject Focus: β = −0.29, *SE* = 0.06, *t* = −4.94). This difference may be due to the fact that Dutch and Chinese participants initiated speaking at a different point relative to their progress with sentence preparation. However, we cannot conclude what this difference is due to in the current experiments, so it remains an interesting question for future cross-linguistic research.

^3^To verify whether this difference across experiments was due to differences in the syntax of wh-questions in Dutch and Chinese rather than to item differences, we also examined the timecourse of formulation in Experiment 1 (Dutch) for the subset of 43 pictures that were used in both experiments. The same pattern was observed for the smaller dataset as for the larger dataset reported in Experiment 1: Dutch speakers directed their gaze to the object character preferentially only approximately 800 ms after picture onset.
